# LOLA: enrichment analysis for genomic region sets and regulatory elements in R and Bioconductor

**DOI:** 10.1093/bioinformatics/btv612

**Published:** 2015-10-27

**Authors:** Nathan C. Sheffield, Christoph Bock

**Affiliations:** ^1^CeMM Research Center for Molecular Medicine of the Austrian Academy of Sciences,; ^2^Department of Laboratory Medicine, Medical University of Vienna, 1090 Vienna, Austria and; ^3^Max Planck Institute for Informatics, 66123 Saarbrücken, Germany

## Abstract

**Summary:** Genomic datasets are often interpreted in the context of large-scale reference databases. One approach is to identify significantly overlapping gene sets, which works well for gene-centric data. However, many types of high-throughput data are based on genomic regions. Locus Overlap Analysis (LOLA) provides easy and automatable enrichment analysis for genomic region sets, thus facilitating the interpretation of functional genomics and epigenomics data.

**Availability and Implementation:** R package available in Bioconductor and on the following website: http://lola.computational-epigenetics.org.

**Contact**: nsheffield@cemm.oeaw.ac.at or cbock@cemm.oeaw.ac.at

Many types of biological data can be interpreted by comparing them to reference databases and searching for interesting patterns of enrichment and depletion. A particularly successful approach focuses on identifying significant overlap between gene sets. To this end, a gene set of interest is compared with a large compendium of existing gene sets with biological annotations, and the observed patterns of overlap are used for interpreting the new gene set. This type of analysis is exemplified by the popular GSEA tool (Subramanian *et al.*, 2005), and it relies on existing gene set annotation databases such as Gene Ontology, KEGG Pathways and MSigDB.

Although gene set analysis has been pivotal for making connections between diverse types of genomic data, this method suffers from one major limitation: it requires gene-centric data. This is becoming increasingly limiting as our understanding of gene regulation advances. Genes are no longer viewed as monolithic building blocks but as multifaceted elements with alternative splicing and alternative promoters, as well as various types of non-coding, antisense and regulatory transcripts. Furthermore, it has become evident that gene expression and chromatin organization are controlled by 100 000s of enhancers and other functional elements, which are often difficult to map to gene symbols. The increasing emphasis on genomic region sets has been propelled by next generation sequencing—a technology that produces data most naturally analyzed in the context of genomic regions, for example as peaks and segmentations. Driven by projects such as ENCODE (Encyclopedia of DNA Elements) and IHEC (International Human Epigenome Consortium), the research community has established large catalogs of regulatory elements and other genomic features across many cell types.

Here, we present an R/Bioconductor package called LOLA (*Locus Overlap Analysis*) for enrichment analysis based on genomic regions. LOLA builds upon analytical concepts that we developed and applied in previous work (Bock *et al.*, 2012; Farlik *et al.*, 2015; Tomazou *et al.*, 2015), and our software makes genomic region set analysis fast and easy for any species with an annotated reference genome. LOLA complements existing tools for gene set analysis (Khatri *et al.*, 2012), tools that convert gene sets into genomic loci such as GREAT (McLean *et al.*, 2010) and the ChIP-Seq Significance Tool (Auerbach *et al.*, 2013), and other related tools including GenometriCorr (Favorov *et al.*, 2012), Genomic HyperBrowser (Sandve *et al.*, 2013), EpiGRAPH (Bock *et al.*, 2009), genomation (Akalin *et al.*, 2014), i-CisTarget (Imrichova *et al.*, 2015), Genome Track Analyzer (Kravatsky *et al.*, 2015), ColoWeb (Kim *et al.*, 2015) and ReMap (Griffon *et al.*, 2015). Key features of LOLA are its integration with R and Bioconductor; a command-line interface supporting automated data processing; compatibility with high-throughput pipelines as well as interactive scripting in R; fast runtime even for very large region lists and reference databases; a comprehensive core database of regulatory elements; and convenient support for users to create custom reference databases.

Each LOLA analysis is based on three components (Fig. 1A): (i) The query set—one or more lists of genomic regions to be tested for enrichment; (ii) a region universe—the background set of regions that could potentially have been included in the query set; and (iii) a reference database of genomic region sets that are to be tested for overlap with the query set. LOLA includes a core reference database assembled from public data, including, for example, the CODEX database (Sanchez-Castillo *et al.*, 2014) and cross-tissue annotation of DNase hypersensitivity (Sheffield *et al.*, 2013). Alternatively or in addition, users can create problem-specific custom regions sets. To build a custom reference database, it is sufficient to collect text files with genomic coordinates (BED files) into a folder and to annotate them with descriptive names.

**Fig. 1.**
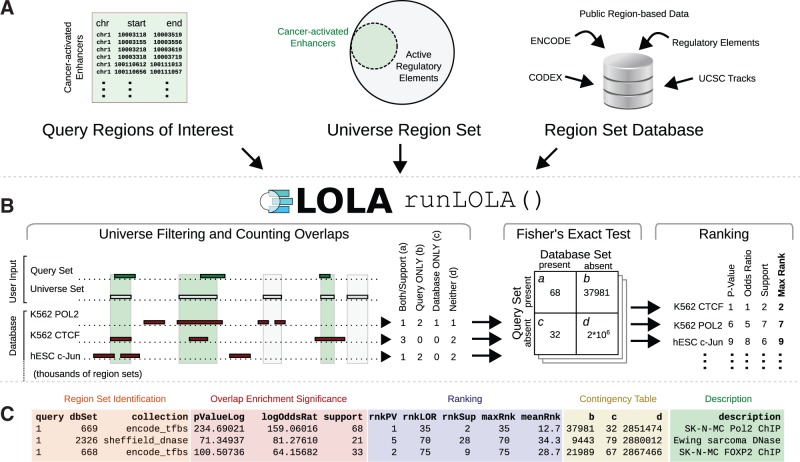
LOLA workflow and results. (**A**) Query sets, universe set and reference database are loaded into R. (**B**) LOLA identifies overlaps, calculates enrichment and ranks the results. (**C**) Example of ranked LOLA enrichment results obtained by runLOLA()

## A simple example

Here we analyze a set of the top-100 strongest EWS-FLI1 binding peaks from a previous study (Tomazou *et al.*, 2015) and assess their overlap with public data. The query set and the LOLA core database are available from the LOLA website.

queryA = readBed(“setA.bed”)

activeDHS = readBed(“activeDHS_universe.bed”)

lolaDB = loadRegionDB(“LOLACore/hg19”)

result = runLOLA(queryA, activeDHS, lolaDB)

result[1:3,] # View top results

LOLA identifies all genomic regions from a query set that overlap with each region set in the reference database. This analysis is performed against a user-specified region universe, which is defined as the set of regions that could, in principle, have been included in the query set (e.g. subject to coverage constraints of the assay that was used to identify the query regions). By default, a single shared base pair is sufficient for regions to count as overlapping, but a stricter criterion can be chosen by the user. Next, considering each region as independent, LOLA uses Fisher’s exact test with false discovery rate correction to assess the significance of overlap in each pairwise comparison (Fig. 1B). The resulting rank score for each region set is then computed by assigning it the worst (max) rank among three measures: *P*-value, log odds ratio and number of overlapping regions. This ranking system emphasizes overlaps that do well on all three measures, and it tends to prioritize biologically relevant associations (Assenov *et al.*, 2014). Results are returned as a *data.table* object (Fig. 1C), providing a powerful interface to sort, explore, visualize and further process the results. In our example, the top hits accurately identify Ewing sarcoma specific regulatory elements.

LOLA implements several helper functions to explore and export the results. All functions are described on the LOLA website with vignettes illustrating the basic and advanced features. In particular, a tutorial on manipulating the universe region set helps with configuring the most biologically relevant comparisons. Furthermore, the *buildRestrictedUniverse()* function automatically builds a universe based on query sets and can be used to test two region sets for differential enrichment against a reference database.

LOLA facilitates large-scale comparisons by using optimized code for storing region sets and running vector calculations with the *data.table* (Dowle *et al.*, 2015) and *GenomicRanges* packages (Lawrence *et al.*, 2013). It also uses database caching and multiple CPUs to speed up the analysis. These optimizations make LOLA analyses fast and memory-efficient, completing within a few minutes on a standard desktop computer.

Gene sets are sometimes regarded as a universal language connecting genes, diseases and drugs. We anticipate that sets of genomic regions can similarly connect diverse types of genome, epigenome and transcriptome data to identify relevant associations in large datasets, thereby leveraging the broad investment into large-scale functional genomics and epigenomics for biological discovery. Such analyses can now be done easily and efficiently using LOLA.

## References

[btv612-B1] Akalin A. (2014) Genomation: a toolkit to summarize, annotate and visualize genomic intervals. Bioinformatics, 31, 1127–1129. 25417204 10.1093/bioinformatics/btu775

[btv612-B2] Assenov Y. (2014) Comprehensive analysis of DNA methylation data with RnBeads. Nat. Methods, 11, 1138–1140. 25262207 10.1038/nmeth.3115 PMC4216143

[btv612-B3] Auerbach R.K. (2013) Relating genes to function: identifying enriched transcription factors using the ENCODE ChIP-Seq significance tool. Bioinformatics, 29, 1922–1924. 23732275 10.1093/bioinformatics/btt316 PMC3712221

[btv612-B4] Bock C. (2009) EpiGRAPH: user-friendly software for statistical analysis and prediction of (epi-) genomic data. Genome Biol., 10, R14. 19208250 10.1186/gb-2009-10-2-r14 PMC2688269

[btv612-B5] Bock C. (2012) DNA methylation dynamics during in vivo differentiation of blood and skin stem cells. Mol. Cell, 47, 633–647. 22841485 10.1016/j.molcel.2012.06.019 PMC3428428

[btv612-B6] Dowle M. (2015) data.table: extension of data.frame. R package version 1.9.6.

[btv612-B7] Farlik M. (2015) Single-cell DNA methylome sequencing and bioinformatic inference of epigenomic cell-state dynamics. Cell Rep., 10, 1386–1397. 25732828 10.1016/j.celrep.2015.02.001 PMC4542311

[btv612-B8] Favorov A. (2012) Exploring massive, genome scale datasets with the GenometriCorr package. PLoS Comput. Biol., 8, e1002529. 22693437 10.1371/journal.pcbi.1002529 PMC3364938

[btv612-B9] Griffon A. (2015) Integrative analysis of public ChIP-seq experiments reveals a complex multi-cell regulatory landscape. Nucleic Acids Res., 43, e27. 25477382 10.1093/nar/gku1280 PMC4344487

[btv612-B10] Imrichová H. (2015) i-cisTarget 2015 update: generalized cis-regulatory enrichment analysis in human, mouse and fly. Nucleic Acids Res., 43 W1, W57–W64. 25925574 10.1093/nar/gkv395 PMC4489282

[btv612-B11] Khatri (2012) Ten years of pathway analysis: current approaches and outstanding challenges. PLoS Comput. Biol., 8, e1002375. 22383865 10.1371/journal.pcbi.1002375 PMC3285573

[btv612-B12] Kim R. (2015) ColoWeb: a resource for analysis of colocalization of genomic features. BMC Genomics, 16, 1345. 10.1186/s12864-015-1345-3 PMC4364483 25887597

[btv612-B13] Kravatsky Y.V. (2015) Genome-wide study of correlations between genomic features and their relationship with the regulation of gene expression. DNA Res., 22, 109–119. 25627242 10.1093/dnares/dsu044 PMC4379982

[btv612-B14] Lawrence M. (2013). Software for computing and annotating genomic ranges. PLoS Comput. Biol., 9, e1003118. 23950696 10.1371/journal.pcbi.1003118 PMC3738458

[btv612-B15] McLean C.Y. (2010) GREAT improves functional interpretation of cis-regulatory regions. Nat. Biotechnol., 28, 495–501. 20436461 10.1038/nbt.1630 PMC4840234

[btv612-B16] Sandve (2013). The Genomic HyperBrowser: an analysis web server for genome-scale data. Nucleic Acids Res., 41, W133–W141. 23632163 10.1093/nar/gkt342 PMC3692097

[btv612-B17] Sanchez-Castillo M. (2014) CODEX: a next-generation sequencing experiment database for the haematopoietic and embryonic stem cell communities. Nucleic Acids Res., 43, D1117–D1123. 25270877 10.1093/nar/gku895 PMC4384009

[btv612-B18] Sheffield N.C. (2013) Patterns of regulatory activity across diverse human cell types predict tissue identity, transcription factor binding, and long-range interactions. Genome Res., 23, 777–788. 23482648 10.1101/gr.152140.112 PMC3638134

[btv612-B19] Subramanian A. (2005) Gene set enrichment analysis: a knowledge-based approach for interpreting genome-wide expression profiles. Proc. Natl Acad. Sci. U.S.A., 102, 15545–15550. 16199517 10.1073/pnas.0506580102 PMC1239896

[btv612-B20] Tomazou E.M. (2015) Epigenome mapping reveals distinct modes of gene regulation and widespread enhancer reprogramming by the oncogenic fusion protein EWS-FLI1. Cell Rep., 10, 1082–1095. 25704812 10.1016/j.celrep.2015.01.042 PMC4542316

